# Inventory of European Sea Bass (*Dicentrarchus labrax*) sncRNAs Vital During Early Teleost Development

**DOI:** 10.3389/fgene.2019.00657

**Published:** 2019-07-25

**Authors:** Elena Sarropoulou, Elizabet Kaitetzidou, Nikos Papandroulakis, Aleka Tsalafouta, Michalis Pavlidis

**Affiliations:** ^1^Institute of Marine Biology, Biotechnology and Aquaculture, Hellenic Center for Marine Research, Heraklion, Greece; ^2^Department of Biology, University of Crete, Heraklion, Greece

**Keywords:** development, RNAseq, sncRNA, microRNA teleosts, functional genomics

## Abstract

During early animal ontogenesis, a plethora of small non-coding RNAs (sncRNAs) are greatly expressed and have been shown to be involved in several regulatory pathways vital to proper development. The rapid advancements in sequencing and computing methodologies in the last decade have paved the way for the production of sequencing data in a broad range of organisms, including teleost species. Consequently, this has led to the discovery of sncRNAs as well as the potentially novel roles of sncRNA in gene regulation. Among the several classes of sncRNAs, microRNAs (miRNAs) have, in particular, been shown to play a key role in development. The present work aims to identify the miRNAs that play important roles during early European sea bass (*Dicentrarchus labrax*) development. The European sea bass is a species of high commercial impact in European and especially Mediterranean aquaculture. This study reports, for the first time, the identification and characterization of small RNAs that play a part in the 10 developmental stages (from morula to all fins) of the European sea bass. From 10 developmental stages, more than 135 million reads, generated by next-generation sequencing, were retrieved from publicly available databases as well as newly generated. The analysis resulted in about 2,000 sample grouped reads, and their subsequently annotation revealed that the majority of transcripts belonged to the class of miRNAs followed by small nuclear RNAs and small nucleolar RNAs. The analysis of small RNA expression among the developmental stages under study revealed that miRNAs are active throughout development, with the main activity occurring after the earlier stages (morula and 50% epiboly) and at the later stages (first feeding, flexion, and all fins). Furthermore, investigating miRNAs exclusively expressed in one of the stages unraveled five miRNAs with a higher abundance only in the morula stage (miR-155, miR-430a, d1, d2, and miR-458), indicating possible important key roles of those miRNAs in further embryonic development. An additional target search showed putative miRNA-mRNA interactions with possible direct and indirect regulatory functions of the identified miRNAs.

## Introduction

Non-coding RNAs (ncRNAs) are involved in several different regulatory pathways. In the last decade, their importance has been demonstrated in a broad range of organisms, including teleost species (Herkenhoff et al., 2018). Several types of non-coding regulatory RNAs have been identified, chief among them being long ncRNAs and small ncRNAs (sncRNAs). The major classes of sncRNAs are short interfering RNAs, piwi acting RNAs, small nuclear RNAs (snRNA), small nucleolar RNAs (snoRNA), and microRNAs (miRNAs; Labbé et al., 2017). The majority of studies investigated the role of miRNA, which is a highly conserved class of small regulatory RNAs known to act at the translational level mainly by repressing protein production (Gavery and Roberts, 2017). The rapid advancements in sequencing and computing methodologies have significantly enhanced the discovery of new miRNAs not only in humans and model species, such as the zebrafish (*Danio rerio*; Mishima, 2012), but also in non-model species, such as the Atlantic halibut (*Hippoglossus hippoglossus*) (Bizuayehu et al., 2012a) and the catfish (*Ictalurus punctatus*) (Barozai, 2012). Besides their functional importance in diverse biological processes, it has also been shown that miRNAs are essential for vertebrate development (Wienholds and Plasterk, 2005). In teleosts, the importance of miRNA in modulating gene expression during development has been reported in various studies evaluating both model and non-model fish species. The first miRNAs with a regulative function in fish development have been described in zebrafish (Kloosterman et al., 2004). The dynamics of miRNA expression during early ontogeny in non-model fish species, such as the Atlantic halibut (Bizuayehu et al., 2012b), turbot (*Scophthalmus maximus*); (Robledo et al., 2017), and Senegalese sole (*Solea senegalensis*; Campos et al., 2014), have also been reported.

The European sea bass (*Dicentrarchus labrax*) is a species of high commercial impact whose industrial production is steadily growing. Efforts have also been made to intensify fish cultivation and target faster growth rates and better feed conversion ratios. Consequently, several objectives such as the larval survival rate, alternative feeds, and disease resistance are of significant importance to the industry. The embryonic and larval stages are part of the most important periods to ensure high performance and superior quality in the following developmental phases of the life cycle, particularly for fish in captivity (Varsamos et al., 2006; Pittman et al., 2013; Tsalafouta et al., 2015). Over the last decade, these economic interests have directed increased research efforts in the rearing of European sea bass, including the significant enrichment of the molecular toolbox for the study of this species. Today, besides several transcriptome datasets (Sarropoulou et al., 2009; Sarropoulou et al., 2012; Pinto et al., 2017), the whole genome sequence of the European sea bass (Kuhl et al., 2010; Tine et al., 2014) as well as single nucleotide polymorphism markers, genetic linkage maps (Chistiakov et al., 2008; Guyon et al., 2010), and radiation hybrid maps (Guyon et al., 2010) are available. However, scarce information has been published concerning ncRNAs (Kaitetzidou et al., 2015).

The present work aims to identify sncRNAs and their targets that play an important role in the development of the European sea bass. Teleost development can be reflected as a sequence of ongoing morphological changes, whereby the embryonic and larval stages are considered to be the most significant time points in the life cycle of marine fish. In the natural environment and during its embryonic and larval phases, the European sea bass lives in the marine environment, whereas as a juvenile it migrates to coastal zones, estuaries, and lagoons. The European sea bass is therefore considered a euryhaline species with reportedly high adaptation processes during its early life phases. To detect most of the sncRNAs that are important to the European sea bass development and to obtain a list of unique miRNAs, this study analyzed the small RNA transcripts of early development (as described by Kaitetzidou et al., 2015) along with newly generated sequencing reads from three additional later stages. It reported for the first time the identification and characterization of the European sea bass small RNAs during development (from morula to all fins); using target search, it also revealed the putative miRNA–mRNA interactions resulting in the possible direct and indirect regulatory functions of the selected miRNAs, which show differential expression (DE) during development.

## Materials and Methods

All experiments/methods in the present study were performed in accordance with the approved guidelines and regulations from the Hellenic Center for Marine Research (HCMR) Institutional Animal Care and Use Committee following the three Rs (Replacement, Reduction, and Refinement) guiding principles for more ethical use of animals in testing, which was first described by Russell and Burch in 1959 (EU Directive 2010/63). These principles are now followed in many testing establishments worldwide before the initiation of experiments.

### Sampling and RNA Extraction

For the generation of new data from three later developmental stages, samples were collected from i) the first feeding stage (FF), when the mouth opens, the pectoral fins have been developed, and the yolk sac has been completely absorbed; ii) the flexion (FLX) stage, when the notochord flexion has been completed; and iii) the all-fins stage (FINS), when all fins have been established (Pavlidis et al., 2011). All samplings were carried out at the installations of the Institute of Marine Biology, Biotechnology, and Aquaculture (IMBBC), HCMR (Heraklion, Crete, Greece). For each of the newly sampled stages, i.e., FF, FLX, and FINS, three biological replicates were sampled, flash frozen in liquid nitrogen, and transferred to a −80°C ultra-low freezer until miRNA library preparation. For miRNA library construction and sequencing, the same method as described by Kaitetzidou et al. (2015) was followed. In brief, total RNA was extracted from all developmental stages using Nucleospin miRNA Kit for the isolation of small RNA (sncRNA) and mRNA (Macherey-Nagel GmbH & Co. KG, Duren, Germany) according to the manufacturer’s instructions. Larvae were disrupted with mortar and pestle in liquid nitrogen and homogenized in lysis buffer by passing lysate through a 23-gauge (0.64 mm) needle five times. The quantity of RNA was estimated with NanoDrop ND-1000 spectrophotometer (NanoDrop Technologies, Inc., Wilmington, DE, USA) and the quality was further evaluated by agarose (1%) gel electrophoresis and Agilent 2100 Bioanalyzer using RNA Nano Bioanalysis chip.

### miRNA Library Preparation and Sequencing

For all samples, miRNA libraries were prepared according to the manufacturer’s instructions and single-end sequencing was carried out using Illumina sequencing technology platform (Illumina HiSeq 2000) at the Cornell University Core Laboratories Center. The use of multiplex identifier tags for each library allowed the pooling of the samples to be run in one HiSeq 2000 lane. The quality control of all reads was assessed by running Fastqc version 0.10.0 (http://www.bioinformatics.babraham.ac.uk/projects/fastqc).

### Sequence Reads Analysis

Raw sequencing data from seven early developmental stages, i.e., morula (M), 50% epiboly (E), late gastrulation-organogenesis (GO), organogenesis (O), late organogenesis (LO), hatching (HA), and 24 h post-hatching (hph), were retrieved from Kaitetzidou et al. (2015). Sequence reads from a total of 10 developmental stages, i.e., reads obtained from Kaitetzidou et al. as well as the newly generated reads from the FF, FLX, and FINS stages, were quality and adapter trimmed using Trimmomatic software 0.30 (Bolger et al., 2014) and imported into CLC Genomics Workbench version 10.1 (CLC Bio, Aarhus, Denmark). The annotation of unique sncRNAs was performed against Gasterosteus_aculeatus.BROADS1.ncrna as well as against available miRNA annotations of teleosts, humans, and mice in miRBase (Griffiths-Jones et al., 2008; *Cyprinus carpio*, *D. rerio*, *Fugu rubripes*, *I. punctatus*, *Oryzias latipes*, *Petromyzon marinus*, *Salmo salar*, *Tetraodon nigroviridis*, *Gallus gallus*, *H. hippoglossus*, *Homo sapiens*, *Mus musculus*, and *Paralichthys olivaceus*).

### Differential Expression

For DE analysis, transcript counting was carried out with CLC Genomics Workbench 10.1.1 (CLC Bio; https://www.qiagenbioinformatics.com/). For all not yet normalized transcripts, read counts greater than five were considered for DE analysis. For DE sequencing reads, which could not be annotated through the Gasterosteus_aculeatus.BROADS1.ncrna database, additional effort was made by submitting them to publicly available databases of the National Center for Biotechnology Information (NCBI; non-redundant nucleotide and expressed sequence tags). Transcripts per million normalized count values were transformed into a decimal logarithmic scale. Obtained small RNA reads were mapped to the *D. labrax* genome for validation purposes. DE was assessed based on the empirical analysis method (EDGE test) provided within the CLC Genomics Workbench with default parameters. Transcripts were considered significant DE if false discovery rate (FDR) values were below 0.05 and log_2_fold change > 2. To find stage-specific miRNAs, transcripts found only in one of the stages but zero counts in the other were considered as stage-specific miRNA.

### miRNA Target Search

**Figure 1:**
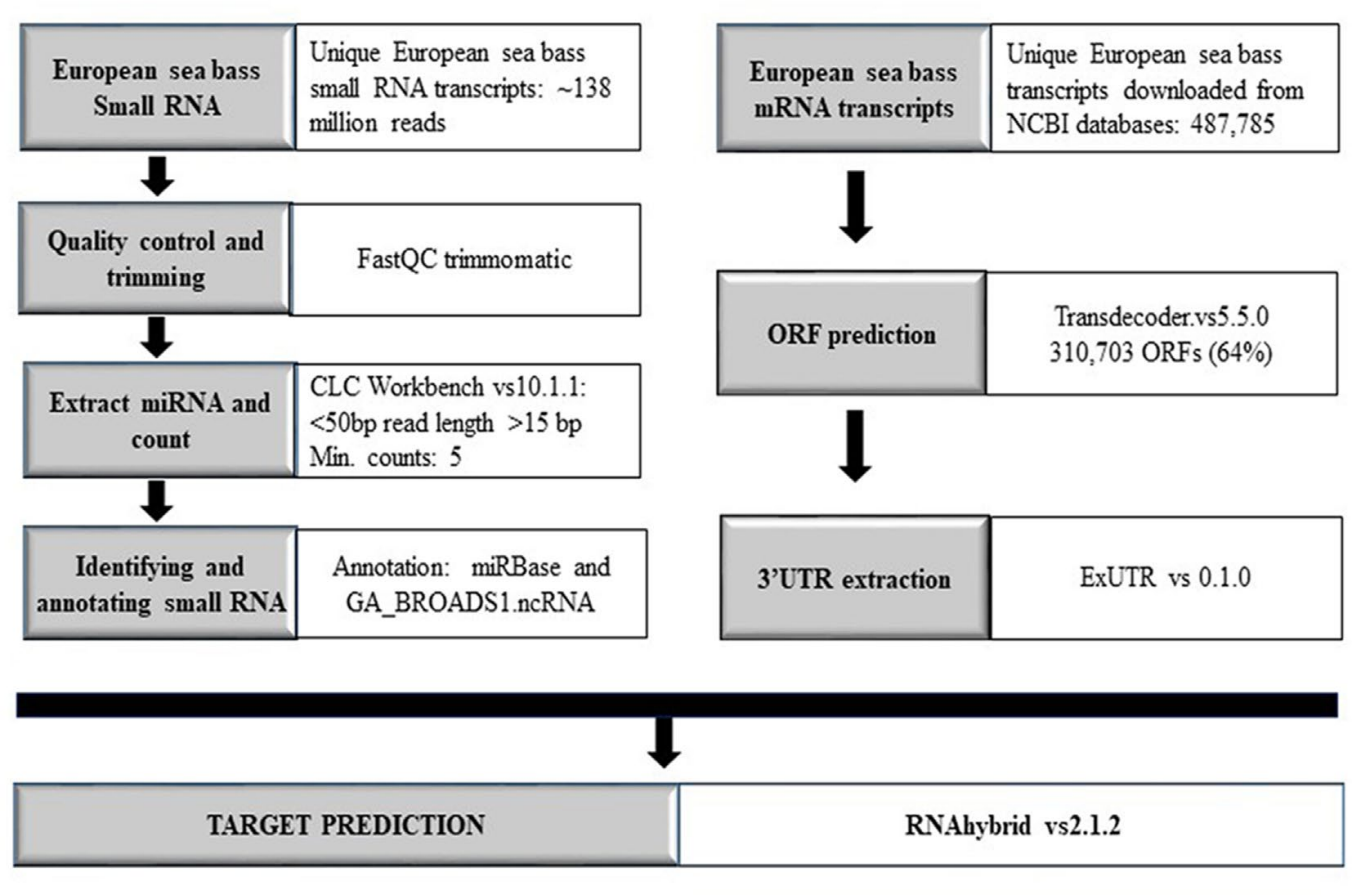
Workflow overview comprising all steps and methods used in the present work. Annotation with miRBase was carried out by applying the databases of *C. carpio*, *D. rerio*, *F. rubripes*, *I. punctatus*, *O. latipes*, *P. marinus*, *S. salar*, *T. nigroviridis*, *G. gallus*, *H. hippoglossus*, *H. sapiens*, *M. musculus*, and *P. olivaceus*.

For target search, the coding sequence information (mRNA) for the European sea bass was extracted from the NCBI nucleotide databases and open reading frame prediction was carried out using Transdecoder version 5.5.0 software program (Haas et al., 2013). 3′ Untranslated regions were extracted by ExUTR version 0.1.0 and RNAhybrid version 2.1.2 (Krüger and Rehmsmeier, 2006) as well as miRanda web tool (Enright et al., 2003) and used for target prediction with the default parameters and an energy threshold of mfe < −30 kcal. The complete workflow is illustrated in **Figure 1**.

### Gene Ontology (GO) Classification and Enrichment Analysis of Target Genes

GO terms were retrieved using the PANTHER classification system (http://www.pantherdb.org/). The chosen analysis type was “Statistical overrepresentation test” and the annotation data set the PANTHER GO-Slim Biological Process data set (PANTHER version 14.1 Released 2019-03-12). The analyzed list consisted of the zebrafish target genes for miR-430 and miR-21 retrieved from TargetScanFish (TargetScanFish, release 6.2, June 2012; Ulitsky et al., 2012). The reference gene list for the test consisted of all genes in the PANTHER database for zebrafish. The test type was Fisher’s exact test with FDR correction. The statistically overrepresented GO terms of miR-430 and miR-21 target genes were illustrated using PANTHER Overlaid Area Chart of Difference (observed vs. expected).

## Results

### Small RNA Libraries from Developmental Stages of the European Sea Bass

**Figure 2:**
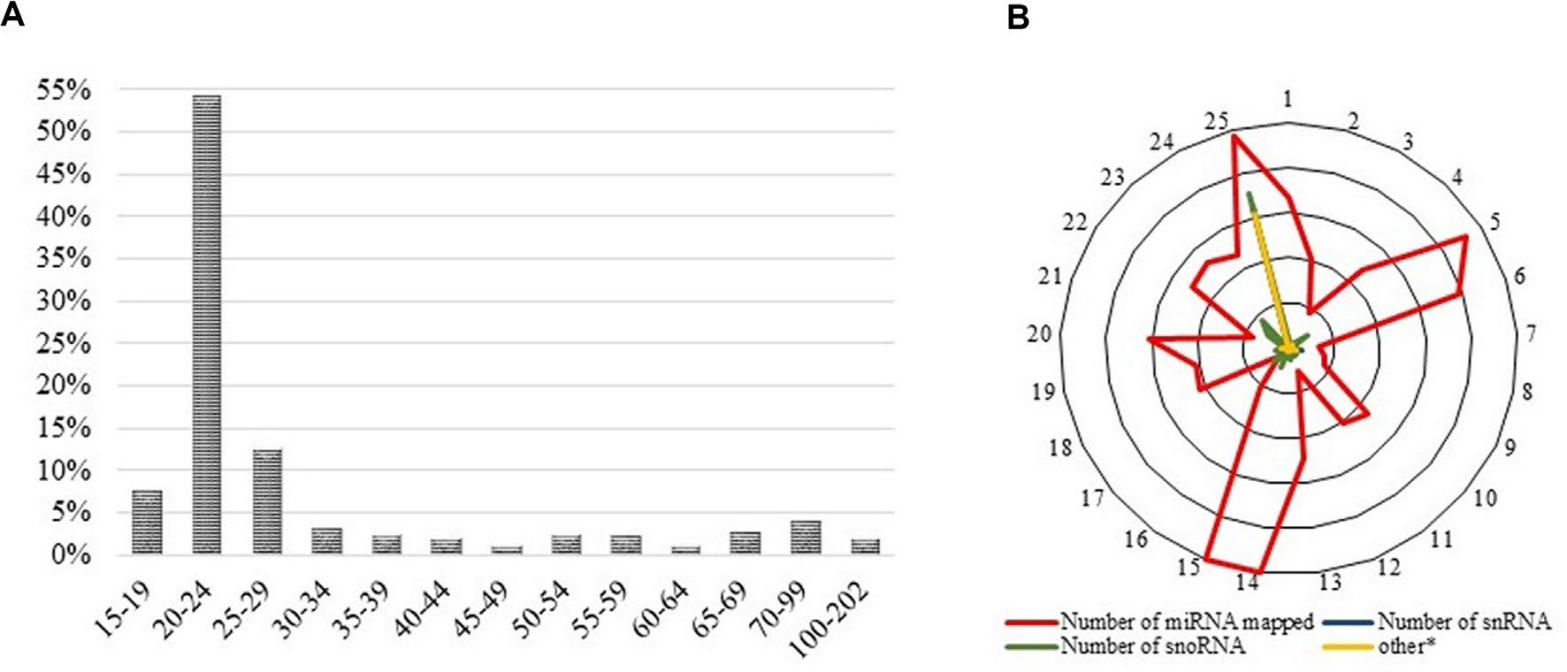
**(A)** Read length distribution of all sequencing reads after trimming and sample grouping. *Y*-axes show the percentage of read counts at a specific read length, whereas *X*-axes show read lengths. **(B)** Successfully mapped small RNA on the available European sea bass (*D. labrax*) genome. The outer ring illustrates the chromosome number, whereas the inner rings present the relative amount of mapped miRNAs.

For samples FF, FLX, and FINS, new miRNA libraries were generated. RNA quality control by DNAnalyzer Nano RNA chip before library construction showed high-quality total RNA with an RIN number > 8 (**Supplemental Figure S1**). Only samples passing this evaluation step were used for next-generation sequencing (NGS). For each sample after trimming, an average of about 7 million reads were obtained and about 40% were annotated. Annotated read counts for each stage were grouped (“sample grouped”) and resulted in an average of 1,380 annotated reads per stage. An overview of sequencing read numbers is listed in **Table 1**. Sample grouped reads of all stages together amounted to 2.115 reads, whereas sequence lengths of 20 to 24 bp were more than 50% (**Figure 2A**). The total number of successfully mapped small RNA (sample grouped) onto the European sea bass genome amounted to 1,524 reads (∼72%; **Supplemental Table S1**) with 1,169 (77%) reads corresponding to miRNA (**Figure 2B**). Raw sequencing reads were submitted to the SRA database of the NCBI under accession numbers PRJNA269278 and PRJNA369460.

**Table 1:** Summary of sequencing results.

Stage	Total raw reads	Total reads after trimming	Mapped to *D. labrax* genome		Annotated reads		Sample grouped
			Mapped reads	% Total raw reads	Annotated reads	% Total raw reads	
**M**	15,998,453	15,498,366	13,958,077	87%	3,118,746	19%	**1,137**
**E**	9,058,134	8,803,908	8,172,363	90%	2,161,068	24%	**1,417**
**GO**	2,850,816	2,774,995	2,692,154	94%	1,245,144	44%	**1,114**
**O**	4,226,240	4,100,025	4,040,160	96%	2,423,464	57%	**1,272**
**LO**	8,569,035	8,359,789	8,177,466	95%	5,684,218	66%	**1,512**
**HA**	4,728,824	4,334,305	3,973,247	84%	1,273,441	27%	**1,200**
**24 hph**	8,768,147	8,302,090	4,917,489	56%	644,598	7%	**1,183**
**FF-1**	8,270,389	7,946,993	6,542,142	79%	3,989,936	48%	**1,532**
**FF-2**	8,417,481	8,053,958	6,913,702	82%	3,139,332	37%	**1,520**
**FF-3**	12,481,129	11,847,863	10,390,994	83%	3,353,378	27%	**1,591**
**FLX-1**	7,180,116	5,976,785	5,269,245	73%	3,422,344	48%	**1,475**
**FLX-2**	6,290,111	5,585,415	5,490,654	87%	2,902,287	46%	**1,390**
**FLX-3**	9,234,101	7,975,749	5,776,120	63%	3,565,313	39%	**1,454**
**FLX-4**	10,374,079	9,354,392	8,737,528	84%	4,222,820	41%	**1,461**
**FINS-1**	6,667,487	5,406,376	4,508,134	68%	2,813,313	42%	**1,346**
**FINS-3**	7,650,256	7,142,228	6,695,388	88%	4,452,559	58%	**1,505**
**FINS-4**	6,719,317	6,176,416	5,853,669	87%	3,227,534	48%	**1,341**
**Average**	**8,087,301**	**7,508,215**	**6,594,620**	**82%**	**3,037,617**	**40%**	**1,379**

### Differentially Expressed miRNAs During Sea Bass Development

**Figure 3:**
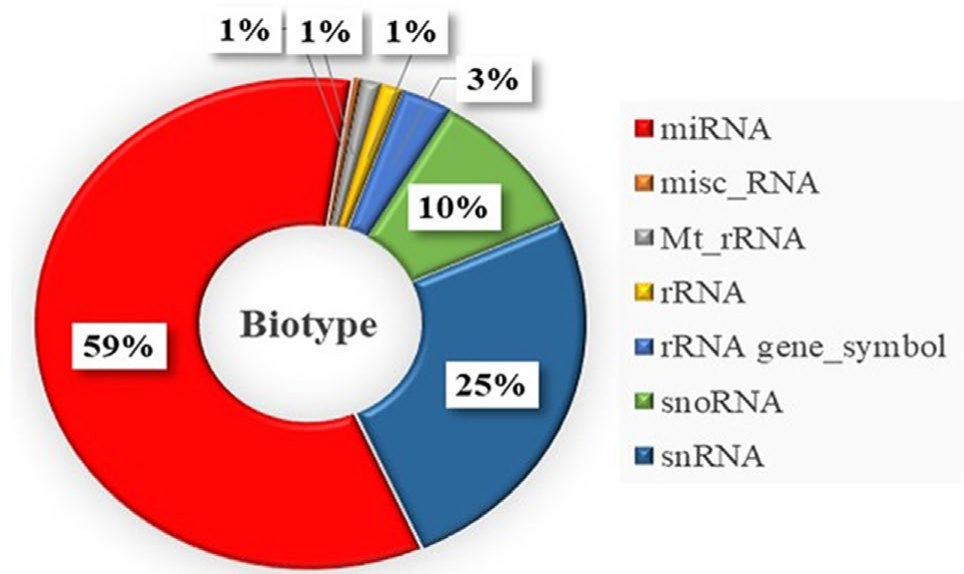
Distribution of obtained small RNA types differentially expressed within the whole sample set. misc_RNA, miscellaneous RNA; Mt_rRNA, mitochondrial rRNA; rRNA gene_Symbol, rRNA gene.

**Figure 4:**
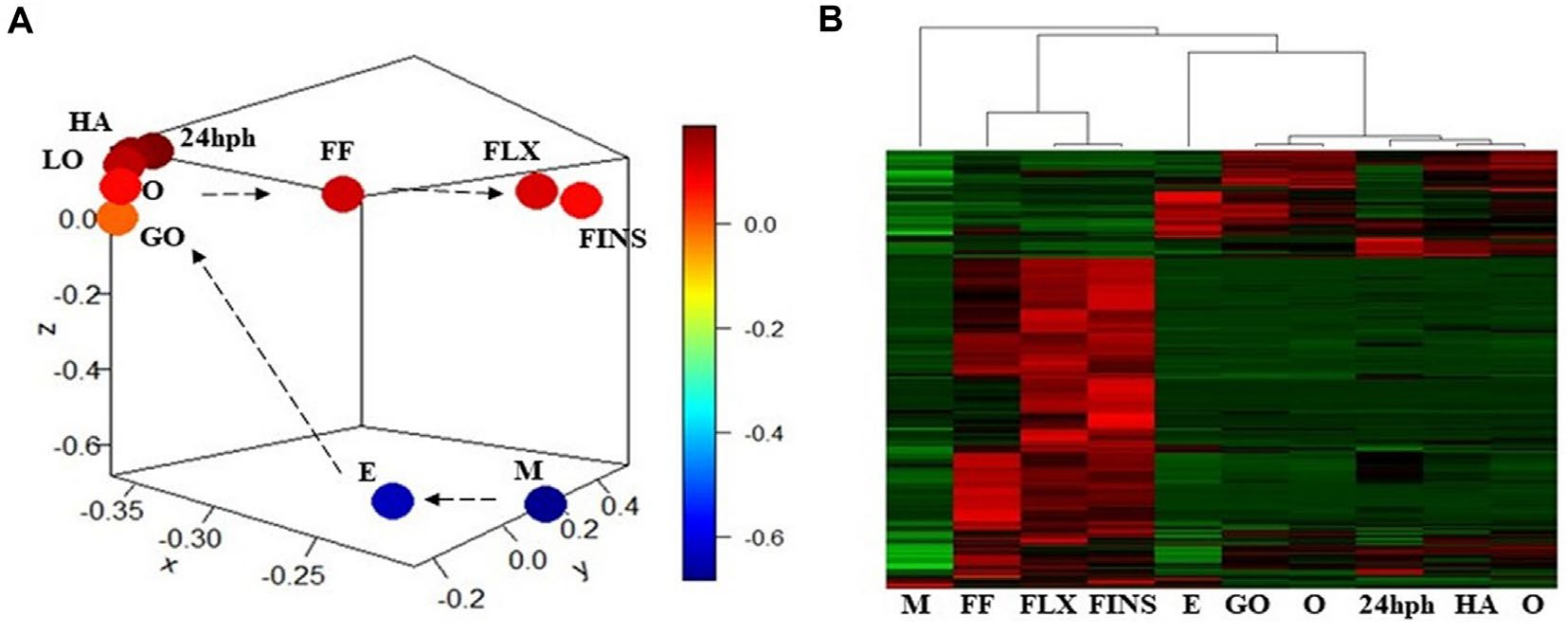
**(A)** Principal component analysis (PCA) plot of normalized mean values of all stages. **(B)** Heatmap of normalized mean values of all significant DE transcripts.

**Figure 5:**
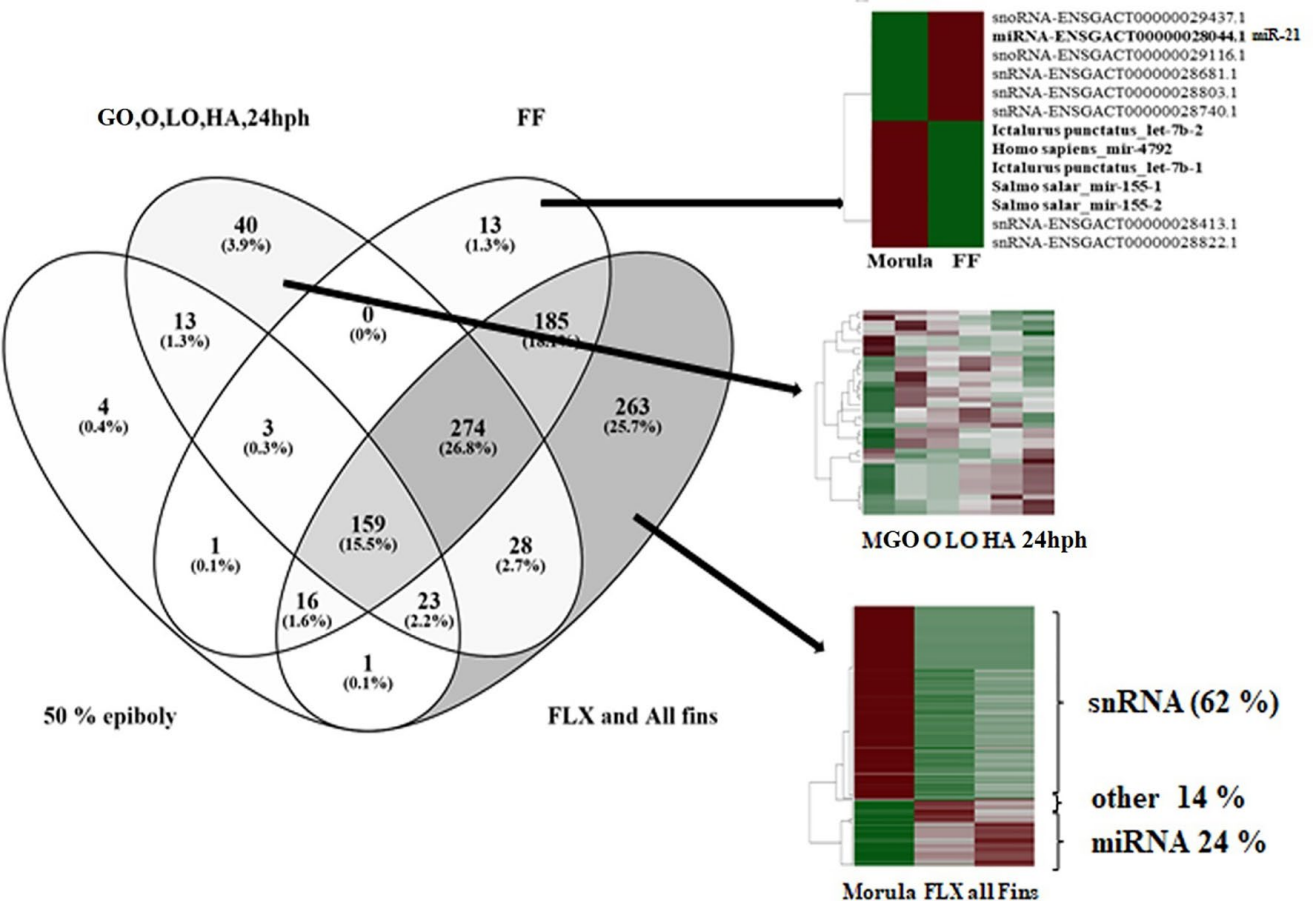
Venn diagram of differentially expressed reads comparing four lists: (1) morula (M) compared to epiboly (E); (2) combined list of the comparisons of stages gastrulation-organogenesis (GO), organogenesis (O), late organogenesis (LO), hatching (HA), and 24 h post-hatching (24 hph) to M; (3) comparison of M to first feeding (FF) stage; and (4) combined list of the comparisons of M to stages flexion (FLX) and all-fins (FINS).

Pair-wise DE analysis was carried out and transcripts with FDR values < 0.05 and log_2_fold change > 2 were considered as DE, resulting in a total of 1,157 annotated reads in at least one comparison among all the 10 stages under study (**Supplemental Table S2**). The majority of transcripts were annotated as miRNA (59%) followed by snRNA (25%) and snoRNA (10%; **Figure 3**). Principal component analysis (PCA) showed that earlier stages separated themselves clearly from the later stages: the FLX and FINS stages (**Figure 4A**). A similar pattern was obtained by hierarchical clustering (**Figure 4B**). According to the PCA plot and the heatmap, the M stage was selected as the reference stage to generate a Venn diagram (**Figure 5**). Furthermore, the comparisons of stages GO, O, LO, HA, and 24 hph to stage M were combined in one list as well as the comparison of stage M to stages FLX and FINS. The Venn diagram showed that the majority of transcripts exclusively expressed belonged to the FLX and FINS stages (263, 26%), with 62% of them classified as snRNA and 24% as miRNA. The list comprising stages GO, O, LO, HA, and 24 hph had 40 (3.9%) unique transcripts (36 miRNAs and four snoRNAs), the FF had 13 (1.3%), and the E stage included only 4 (0.4%) miRNA.

### Stage-Specific miRNAs

Transcripts found only in one of the stages and zero counts in the other were considered as stage-specific miRNA. In the present work, no stage-specific miRNAs were detected for stages E, FLX, FINS, and HA. For the first stage, the M stage, five miRNAs were found, among which three belonged to the miR-430 family. The last studied stage, the FINS stage, also did not show the presence of any uniquely expressed miRNA. Nevertheless, one of the highly abundant small RNAs at this stage was miR-462. Among the few miRNAs regulated between FLX and FINS stages (a total of 9 miRNAs) were hs-miR-7641-1 and hs-miR-7641-2, which were found to be more abundant at the FLX stage.

### Target Search

**Figure 6:**
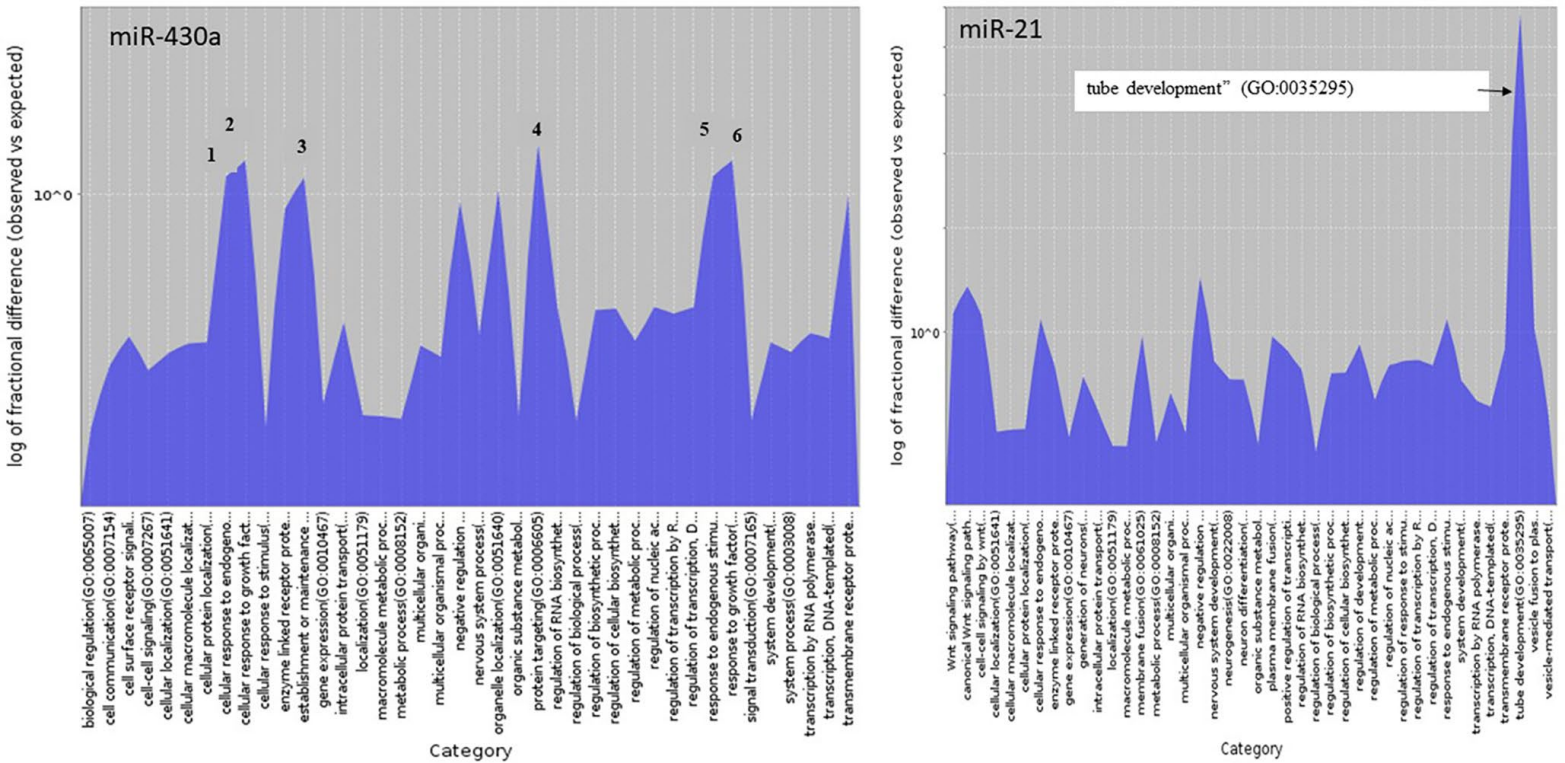
Biological process GO terms of miR-430 and miR-21 target genes statistical overrepresented illustrated using PANTHER Overlaid Area Chart of Difference (observed vs. expected). The numbers of the six most represented GO terms of the miR-430 target genes correspond to **(1)** cellular response to endogenous stimulus, **(2)** cellular response to growth factor stimulus, **(3)** establishment or maintenance of cell polarity, **(4)** protein targeting, **(5)** response to endogenous stimulus, and **(6)** response to growth factor.

miRNA target search in European sea bass, applying positive hybridization scores with an energy threshold < −30.0, was carried out at the gene level for the 14 stage-specific miRNAs (transcript found only in one stage and zero counts in the other stages). The putative targets for the European sea bass, as identified by RNAhybrid, are listed in **Supplemental Table S3**. miRNA target search in zebrafish with miR-430 (stage-specific miRNA) and miR-21 (up-regulated in FF) resulted in specific GO terms illustrated in **Figure 6** and listed in **Supplemental Table S4**.

## Discussion

### Deep Sequencing of European Sea Bass Developmental Stages

Today, NGS has made it possible to assess not only gene expression but also the abundance of small RNA within a given sample. Here, we analyzed more than 135 million reads obtained from 10 developmental stages of the European sea bass comprising NGS data from a previously published work (Kaitetzidou et al., 2015) as well as from the present study. Read numbers varied across the samples, but the numbers of annotated reads, which were “sample grouped,” were alike (**Table 1**), pinpointing the fact that sufficient reads for each sample were obtained. The majority (>50%) of the trimmed and sample grouped reads were 20 to 24 bp long (**Figure 2A**). Including the transcripts read lengths of 15 to 19 bp as well as 25 to 29 bp resulted in more than 70% of the sample grouped reads, demonstrating that the libraries were significantly enriched with miRNAs.

### Small RNA Analysis

Two main approaches exist for analyzing the small RNA reads obtained by NGS. Either the different types of small RNAs in the data are counted or the read counts are first mapped to an appropriately annotated reference genome and then submitted to publicly available databases for annotation purposes. The first approach does not require an annotated genome for mapping, and small RNAs not mapping to the reference genome (due to gaps for instance) can still be measured. In the present study, the first approach was implemented to detect all possible small RNAs obtained by NGS. Nevertheless, with the aim of locating the obtained small RNAs within the genome, reads were mapped to the European sea bass genome. Notably, the majority of miRNAs mapped to four linkage groups (LG5, LG14, LG15, and LG25; **Figure 2B**). Other studies, in about 20 species, have also investigated the detailed chromosome-specific location of miRNA genes. The authors of these studies showed that, in all species, certain chromosomes accumulated a higher number of miRNAs and that miRNAs involved in specific diseases also accumulated in a specific location (e.g., Ghorai and Ghosh, 2014). The aforementioned study by Ghorai and Gosh (Ghorai and Ghosh, 2014) also included three teleost species: the zebrafish, medaka (*O. latipes*), and tetraodon (*T. nigroviridis*). Although miRNAs clearly accumulated in specific chromosomes in zebrafish and medaka, in tetraodon, no clear preference was shown. Nevertheless, the authors listed the top four highest numbers of miRNA genes containing chromosomes, which were chromosomes 7, 4, 14, and 17 for medaka and chromosomes 2, 10, 9, and 13 for tetraodon (chromosome numbers ordered by the highest miRNA counts found). Medaka chromosome 7 is the homolog group to tetraodon 9 (Sarropoulou et al., 2008) and corresponds to the European sea bass LG25, which also showed an abundance of miRNA in the present study (**Figure 2B**). The European sea bass LG14 corresponds to medaka chromosome 14 (third highest number of miRNA in medaka) and LG15 to medaka chromosome 21 (fifth highest number of miRNA in medaka) and tetraodon 2 (highest number of miRNA in tetraodon). Of the four LGs with enriched miRNA counts (LG5, LG14, LG15 and LG25), only European sea bass LG5 did not match with any of the chromosomes with enriched miRNA counts in medaka and tetraodon. In humans, it has been shown that clustered miRNAs are involved in specific pathways or, in particular, cell functions and that clustered miRNAs may be maintained through evolution (Guo et al., 2014). In teleosts, this aspect has not yet been investigated and may be the objective of future studies.

### Differentially Expressed miRNAs During Sea Bass Development

DE miRNAs showed that the investigated earlier stages (i.e., M and E) and the latest stages (i.e., FLX and FINS) separated themselves clearly from the other stages (**Figure 4A**). Therefore, a first comparison to the M stage was carried out, grouping together stages GO, O, LA, HA, and 24 hph as well as stages FLX and FINS (**Figure 5**).

### Between Morula and 50% Epiboly

A total of 220 transcripts were found to be DE between the two earliest stages studied within the present work. Of the 220 transcripts, 165 (75%) were annotated as miRNAs, and 70 miRNAs were found in higher abundance in the E stage. The other 95 miRNAs were detected to be more abundant in the M stage. Four transcripts were exclusively DE in the M vs. E comparison (**Figure 5**). All of them were found in higher abundance in the M stage and appeared to belong to the miR-30a family (miR-30a-1, miR-30a-2, miR-30a-3, and miR-30a-4). For comparison, in *Xenopus*, studies have shown that pri-miRNAs of miR-30a are active only during maternal stages and that no zygotic transcription could be detected within the studied stages (Nepal et al., 2015). Higher miR-30a abundance during the blastula period in *Xenopus* was also shown to be linked to early neural crest development (Ward et al., 2018). In contrast, the highest miRNA fold change between the M and E stages was found for miR-196b with 0 and 1.111 copy numbers, respectively. In zebrafish, research has shown that miR-196 is involved in the regulation of axial patterning and pectoral appendage initiation and that the first appearance of miR-196b has been seen to be later than it was detected in the present study, i.e., at the bud stage (He et al., 2011).

### Between Morula, 50% Epiboly and Gastrulation-Organogenesis, Organogenesis, Late Organogenesis, Hatching, and 24 Hours Post-Hatching

According to the PCA plot, the stages from GO to 24 hph are grouped closely together in comparison to the other stages. Therefore, in the Venn diagram, DE transcripts between those stages and the M stage were considered as one list (**Figure 5**). Here, a total of 40 transcripts, comprising 36 miRNAs and 4 snoRNAs, were found to be exclusively DE. The fact that 90% of the reads exclusively found in the M-GO comparison to 24 hph stages are annotated as miRNA may indicate that miRNAs are more active during early development. Similar findings were described in zebrafish, where an increase of miRNA expression was observed as early development proceeded (Yao et al., 2014).

### Between Morula and First Feeding Stage

Likewise, 13 transcripts (6 miRNAs, 2 snoRNAs, and 5 snRNAs) were found to be exclusively present in the FF stage. Five of the six identified miRNAs, namely miR-4792, let-7b-1, let-7b-2, miR-155-1, and miR-155-2, showed higher abundance in the M stage than in the FF stage (**Figure 5**). Whereas let-7 is a well-studied miRNA belonging to the first founding miRNAs with possible roles in growth development (Kloosterman et al., 2004; Zhao et al., 2017), both miR-155 and miR-4792 have been examined less. Of the remaining miRNAs, miR-21 (ENSGACT00000028044.1) showed higher expression levels in the FF stage. It is also among the first miRNAs to have been identified (Kim, 2005) and has been found to be involved in various biological processes, including development (Kumarswamy et al., 2011). In zebrafish, miR-21 expression was found at very early developmental stages (Chen et al., 2005), whereas, in rainbow trout, miR-21 was suggested to play an important role in degrading maternally inherited mRNAs (Ramachandra et al., 2008). Enrichment analysis of target genes in zebrafish, identified *via* TargetScanFish (Ulitsky et al., 2012), revealed a high number of genes classified in the biological process termed as “tube development” (GO:0035295; **Figure 6**). The formation of tubes such as epithelial and endothelial tubes are of importance in view of gases, liquids, and cell transport. In tilapia (*Oreochromis niloticus*), it has been shown that miR-21 is involved in the modulation of alkalinity stress (Zhao et al., 2016). During European sea bass development, stress, as indicated by water cortisol measurement, was first detected at the FF stage (Tsalafouta et al., 2015). The present finding may indicate that miR-21 in the European sea bass is also involved in mechanisms related to the modulation of stress.

### Between Morula and Flexion/All Fins

The comparison between M and FLX/FINS stages revealed the highest number (263) of exclusively found transcripts. Notably, of the 263 transcripts, only 62 (24%) were classified as miRNA, whereas 164 (62%) reads were annotated as snRNA; all of them were found in higher copy numbers in the M stage. snRNA molecules are known to be an abundant component of eukaryotic cells (Valadkhan and Gunawardane, 2013), their importance for development having already been demonstrated in *Xenopus* nearly four decades ago (Forbes et al., 1983). The exclusively high abundance of snRNA, as illustrated in **Figure 5**, may indicate the importance of studying snRNAs during development in the near future; however, it is beyond the scope of the present work.

### Stage-Specific miRNAs and Their Targets

Annotated miRNAs detected only in one stage and not in the others were found for the M, GO, O, LO, 24 hph, and FF stages. Unique miRNAs found in only one stage and their targets in the European sea bass are listed in **Supplemental Table S3**. For the first stage of the present study (i.e., the M stage), five miRNAs were found; of these, three belonged to the miR-430 family. In zebrafish, it has been shown that the miR-430 family comprised 72 members and that they targeted a large number of maternal mRNAs (Giraldez et al., 2006). The results of miR-430 target search using TargetScanFish in the present study are illustrated in **Figure 6**. Among the significantly enriched biological process categories were the GO terms “establishment or maintenance of cell polarity,” “protein targeting,” “response to growth factor,” “cellular response to growth factor stimulus,” “response to endogenous stimulus,” and “cellular response to endogenous stimulus.” It has been documented in zebrafish that miR-430a directs cell division, which in turn leads to neural tube development (Takacs and Giraldez, 2016); this is also consistent with the findings of this study. Concerning miR-430d, two clusters were found to comprise the mature mirR-430d sequence. These clusters are located on different regions of the genome and appear to fold into the miRNA typical stem-loop (**Supplemental Figure S2**).

The FINS stage, determined by the end of metamorphosis and the start of squamation, did not show the presence of any unique DE miRNA. Nevertheless, one of the highly abundant small RNAs at this stage, compared to the others, was miR-462, which has been linked to growth and muscle development in the blunt snout bream (*Megalobrama amblycephala*; Yi et al., 2013). The authors have found miR-462 to have a higher count number in small-sized fish than in bigger fish. However, in both cases, a high count number was reported. Among the few miRNAs being regulated between stages FLX and FINS (9), hs-miR-7641-1 and hs-miR-7641-2 were found to be more abundant at the FLX stage. miR-7641 in humans is known as a regulator of ribosomal proteins (Reza et al., 2017). For humans, about 3,500 targets have been identified (TargetScanHuman, release 7.2, March 2018). The respective search in zebrafish did not result in any match. Target search using the European sea bass transcriptome identified the gene paralemmin-1 as a putative target for miR-7641-1/miR-7641-2. Paralemmin-1 is also among the 3,500 targets identified for humans and is known to play an important role in filopodia induction and spine maturation (Arstikaitis et al., 2008). In contrast, hhi-miR-7641 (*H. hippoglossus*) was detected in all stages, except in GO and O, with the highest expression of hhi-miR-7641 found at the 24 hph stage.

In conclusion, investigating the miRNA repertoire during the early development of a commercially important fish species such as the European sea bass may contribute to a better understanding of regulatory processes during early ontogenesis. In the present study, we identified 2,115 ncRNA transcripts in the European sea bass; of these, 684 were annotated as miRNA. Distinct ncRNA expression profiles of 10 developmental stages were recognized and stage-specific miRNA were identified. Putative targets were also detected to provide first insights into miRNA involvement during early development.

## Data Availability

The datasets generated for this study can be found in NCBI SRA database, PRJNA269278 and PRJNA369460.

## Ethics Statement

All experiments/methods in the present study were performed in accordance with the approved guidelines and regulations from the HCMR institutional animal care and use committee following the three Rs (Replacement, Reduction, Refinement) guiding principles for more ethical use of animals in testing, first described by Russell and Burch in 1959 (EU Directive 2010/63). These principles are now followed in many testing establishments worldwide prior to initiation of experiments.

## Author Contributions

ES conceived the study, drafted the manuscript, and performed the bioinformatics analyses. EK carried out RNA extractions and validation and helped in the miRNA work. AT and NP conceived the sampling and collected them for further analysis. MP initiated the study, critically revised the manuscript, and approved the final version.

## Funding

Financial support for this study has been provided by the Ministry of Education and Religious Affairs, under the Call “ARISTEIA I” of the National Strategic Reference Framework 2007–2013 (ANnOTATE), co-funded by the EU and the Hellenic Republic through the European Social Fund, the European Union Seventh Framework Program (FP7 2010–2014) under the grant agreement no 265957 (CopeWell).

## Conflict of Interest Statement

The authors declare that the research was conducted in the absence of any commercial or financial relationships that could be construed as a potential conflict of interest.

## Supplementary Material

Supplemental Figure S1 Total RNA extraction was evaluated on an RNA Nano Bioanalysis chip (Agilent) as well as by gel electrophoreses. Only samples passing this evaluation step as presented here were used for NGS. (A) Total RNA. (B) Small RNA.

Supplemental Figure S2 miR-430d sequences along with their identified stem-loop.

Supplemental Table S1 Identified small RNAs mapped onto the European sea bass genome with threshold e-value of < 0.00005.

Supplemental Table S2 Normalized mean read counts of significant DE transcripts.

Supplemental Table S3 miRNAs found in only one stage and zero counts in the other stages along with their putative target genes, annotation, and accession number.

Supplemental Table S4 GO terms of putative target genes regulated by miR-21 and miR-430.
