# Supplementary material for: Inventory of European Sea Bass (Dicentrarchus labrax) sncRNAs Vital During Early Teleost Development
Source: Front Genet. 2019 Jul 25;10:657. doi: 10.3389/fgene.2019.00657 (PMC6670005; doi:10.3389/fgene.2019.00657)
Supplement: Supplemental Figure S1 — Total RNA extraction was evaluated on an RNA Nano Bioanalysis chip (Agilent) as well as by gel electrophoreses. Only samples passing this evaluation step as presented here were used for NGS. (A) Total RNA. (B) Small RNA. [file Image_1.pdf]

Supplemental Figure S1: Evaluation of total RNA extraction

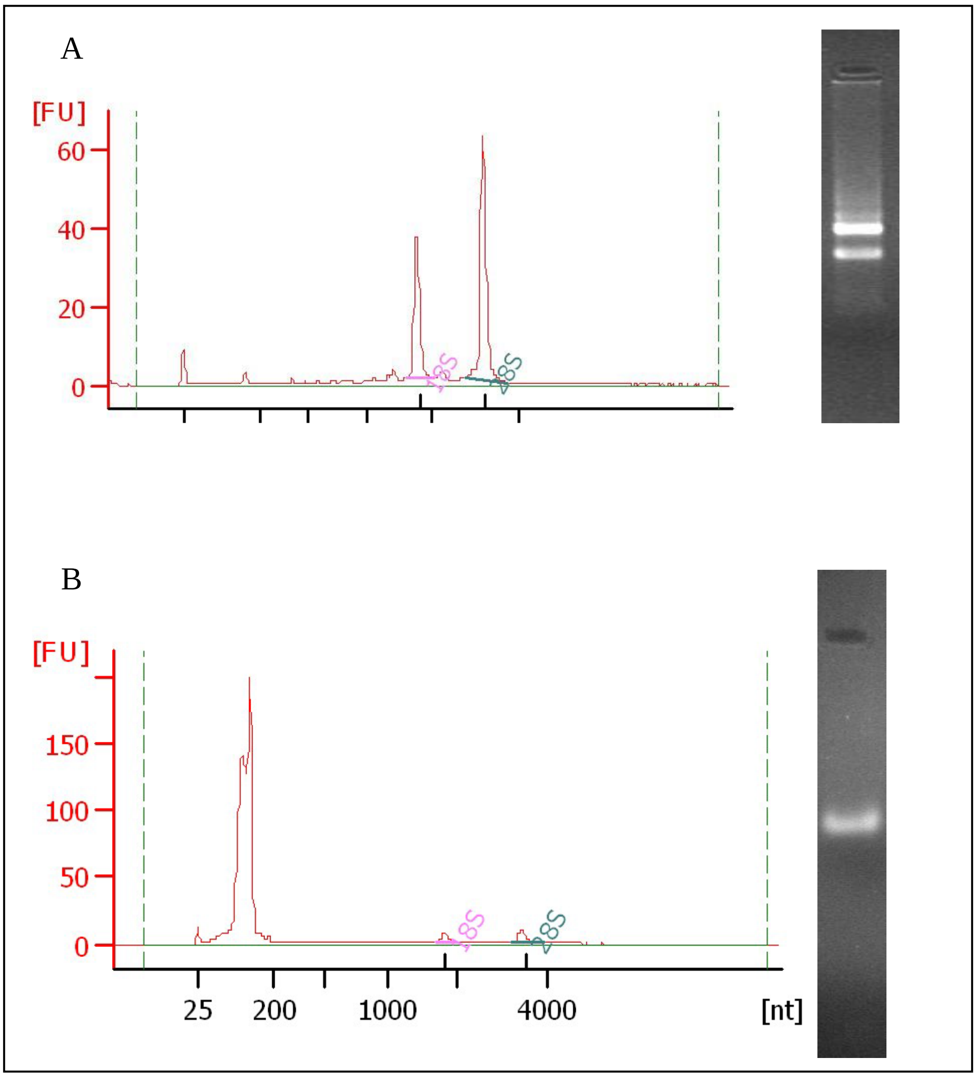

Figure 1: Total RNA extraction were evaluated on a RNA nano BioAnalyzer chip (Agilent) as well as by gel electrophoreses. Only samples passing this evaluation step as presented here were used for NGS sequencing. A: total RNA B: small RNA.
