# Supplementary material for: Inventory of European Sea Bass (Dicentrarchus labrax) sncRNAs Vital During Early Teleost Development
Source: Front Genet. 2019 Jul 25;10:657. doi: 10.3389/fgene.2019.00657 (PMC6670005; doi:10.3389/fgene.2019.00657)
Supplement: Supplemental Figure S2 — miR-430d sequences along with their identified stem-loop. [file Image_2.pdf]

Supplemental figure S3: miR-430d sequences along with their identified stem-loop

groupIV:

>ENSGACT00000028150.1_(Gasterosteus_aculeatus.BROADS1.ncrna)
TCAATGGAATCCAATTCAGATAACCTCAAACTGAGACACTGATGATTCTTCAGTTCATAAGTGCTTCTCTTTGGGGTTGTCTT
>ENSGACT00000028157.1_(Gasterosteus_aculeatus.BROADS1.ncrna)
TCAATGGAATCCAATTCAGATAACCTCAAACTGAGACACTGATGATTCTTCAGTTCATAAGTGCTTCTCTTTGGGGTTGTCTT
>ENSGACT00000028177.1_(Gasterosteus_aculeatus.BROADS1.ncrna)
TCAATGGAATCCAATTCAGATAACCTCAAACTGAGACACTGATGATTCTTCAGTTCATAAGTGCTTCTCTTTGGGGTTGTCTT
>ENSGACT00000028178.1_(Gasterosteus_aculeatus.BROADS1.ncrna)
TCAATGGAATCCAATTCAGATAACCTCAAACTGAGACACTGATGATTCTTCAGTTCATAAGTGCTTCTCTTTGGGGTTGTCTT
>ENSGACT00000028179.1_(Gasterosteus_aculeatus.BROADS1.ncrna)
TCAATGGAATCCAATTCAGATAACCTCAAACTGAGACACTGATGATTCTTCAGTTCATAAGTGCTTCTCTTTGGGGTTGTCTT
>ENSGACT00000028180.1_(Gasterosteus_aculeatus.BROADS1.ncrna)
TCAATGGAATCCAATTCAGATAACCTCAAACTGAGACACTGATGATTCTTCAGTTCATAAGTGCTTCTCTTTGGGGTTGTCTT
>ENSGACT00000028184.1_(Gasterosteus_aculeatus.BROADS1.ncrna)
TCAATGGAATCCAATTCAGATAACCTCAAACTGAGACACTGATGATTCTTCAGTTCATAAGTGCTTCTCTTTGGGGTTGTCTT

Structure 1 Folding bases 1 to 74 of ENSGACT00000028150 1_[Gasterosteus_aculeatus BROAD
Initial ΔG = -26.80

```
        10        20        30        40
UCCAAUUC|           CU  GA    G    UUCU
        AGAUAACCUCAAA  GA  CACU AUGA    \
        UCUGUUGGGGUUU  CU  GUGA UACU    U
U-------^           CU  UC    A    UGAC
          70        60        50
```

scaffold_464:

>ENSGACT00000028271.1_(Gasterosteus_aculeatus.BROADS1.ncrna)
AACACTCTCAGATAACTTCAAATAGAGCCACTGGTGATGATTTGGTTCATAAGTGCTTCTCTTTGGGGTTGTCTTA
>ENSGACT00000028273.1_(Gasterosteus_aculeatus.BROADS1.ncrna)
AACACTCTCAGATAACTTCAAATAGAGCCACTGGTGATGATTTGGTTCATAAGTGCTTCTCTTTGGGGTTGTCTTA
>ENSGACT00000028275.1_(Gasterosteus_aculeatus.BROADS1.ncrna)
AACACTCTCAGATAACTTCAAATAGAGCCACTGGTGATGATTTGGTTCATAAGTGCTTCTCTTTGGGGTTGTCTTA
>ENSGACT00000028275.1_(Gasterosteus_aculeatus.BROADS1.ncrna)
AACACTCTCAGATAACTTCAAATAGAGCCACTGGTGATGATTTGGTTCATAAGTGCTTCTCTTTGGGGTTGTCTTA
>ENSGACT00000028277.1_(Gasterosteus_aculeatus.BROADS1.ncrna)
AACACTCTCAGATAACTTCAAATAGAGCCACTGGTGATGATTTGGTTCATAAGTGCTTCTCTTTGGGGTTGTCTTA
>ENSGACT00000028279.1_(Gasterosteus_aculeatus.BROADS1.ncrna)
AACACTCTCAGATAACTTCAAATAGAGCCACTGGTGATGATTTGGTTCATAAGTGCTTCTCTTTGGGGTTGTCTTA
>ENSGACT00000028281.1_(Gasterosteus_aculeatus.BROADS1.ncrna)
AACACTCTCAGATAACTTCAAATAGAGCCACTGGTGATGATTTGGTTCATAAGTGCTTCTCTTTGGGGTTGTCTTA
>ENSGACT00000028283.1_(Gasterosteus_aculeatus.BROADS1.ncrna)
AACACTCTCAGATAACTTCAAATAGAGCCACTGGTGATGATTTGGTTCATAAGTGCTTCTCTTTGGGGTTGTCTTA

Structure 1 Folding bases 1 to 76 of ENSGACT00000028271 1_[Gasterosteus_aculeatus BROAD
Initial ΔG = -25.50

```
        10        20        30        40
AACACUCUC|           U    C    G    UGAU
         AGAUAACUUCAAA AGAG CACU GUGA    \
         UCUGUUGGGGUUU UCUU GUGA UACU    U
AU-------^           C    C    A    UGGU
             70        60        50
```
