# Supplementary material for: Inventory of European Sea Bass (Dicentrarchus labrax) sncRNAs Vital During Early Teleost Development
Source: Front Genet. 2019 Jul 25;10:657. doi: 10.3389/fgene.2019.00657 (PMC6670005; doi:10.3389/fgene.2019.00657)
Supplement: Supplemental Table S1 — Identified small RNAs mapped onto the European sea bass genome with threshold e-value of < 0.00005. [file Table_1.docx]

| **Chr.** | **Corresponding scaffold (seabass v1.0)** | **Number of miRNA mapped** | **Number of snRNA** | **Number of snoRNA** | **other*** | **Total number of reads mapped** |
| --- | --- | --- | --- | --- | --- | --- |
| 1 | HG916827.1 | 67 | 1 | 4 | 0 | 72 |
| 2 | HG916828.1 | 41 | 0 | 2 | 0 | 43 |
| 3 | HG916829.1 | 18 | 0 | 2 | 1 | 21 |
| 4 | HG916830.1 | 47 | 2 | 3 | 2 | 54 |
| 5 | HG916831.1 | 92 | 1 | 10 | 2 | 105 |
| 6 | HG916832.1 | 78 | 1 | 1 | 0 | 80 |
| 7 | HG916833.1 | 13 | 3 | 2 | 1 | 19 |
| 8 | HG916834.1 | 16 | 6 | 4 | 3 | 29 |
| 9 | HG916835.1 | 17 | 0 | 3 | 0 | 20 |
| 10 | HG916836.1 | 45 | 1 | 4 | 1 | 51 |
| 11 | HG916837.1 | 41 | 1 | 4 | 1 | 47 |
| 12 | HG916838.1 | 11 | 0 | 0 | 0 | 11 |
| 13 | HG916839.1 | 49 | 0 | 5 | 0 | 54 |
| 14 | HG916840.1 | 100 | 0 | 1 | 0 | 101 |
| 15 | HG916841.1 | 100 | 3 | 9 | 1 | 113 |
| 16 | HG916842.1 | 21 | 0 | 2 | 0 | 23 |
| 17 | HG916843.1 | 5 | 2 | 9 | 1 | 17 |
| 18 | HG916844.1 | 43 | 1 | 3 | 1 | 48 |
| 19 | HG916845.1 | 41 | 1 | 6 | 0 | 48 |
| 20 | HG916846.1 | 61 | 4 | 3 | 3 | 71 |
| 21 | HG916847.1 | 17 | 2 | 3 | 0 | 22 |
| 22 | HG916848.1 | 50 | 2 | 11 | 0 | 63 |
| 23 | HG916849.1 | 52 | 1 | 17 | 1 | 71 |
| 24 | HG916850.1 | 47 | 0 | 0 | 0 | 47 |
| 25 | HG916851.1 | 97 | 65 | 71 | 61 | 294 |
| **TOTAL** | | **1169** | **97** | **179** | **79** | **1524** |

**Supplemental table S1. Identified small RNAs mapped onto the European sea bass genome with threshold e-value of < 0.00005**

*other includes ribosomal gene, tRNA, mt trna and miscRNA
